# Does SARS-CoV-2vaccination induce acquired autoimmune discords and inflammatory manifestations?

**DOI:** 10.1016/j.clinsp.2023.100257

**Published:** 2023-07-26

**Authors:** Khadija Bahrini, Emna Hannachi

**Affiliations:** a Research Unit UR17DN05, Military Hospital of Tunis, Tunis El-Manar University, Tunis, Tunisia; b Laboratoire de Parasitologie Médicale, Biotechnologies et Biomolécules; LR11-IPT06; Institut Pasteur de Tunis, Université Tunis El-Manar, Tunis-Belvédère, Tunisia

The Severe Acute Respiratory Syndrome Coronavirus-2 (SARS-CoV-2) has produced an unparalleled setback for the world's economy and health. The most efficient way to reduce infection-related morbidity and mortality is through vaccinations, which are among the most important medical advancements. In response to this pandemic situation, SARS-CoV-2 vaccines are being rapidly developed and most of them have been approved without extensive studies on their side effects and efficacy, compared to traditional vaccines.^1^^,^^2^ In order to prevent infection exacerbation, vaccines are administered to healthy patients, hence side effects and adverse events become quite relevant. As it has been described with other vaccines, many investigations have shown that SARS-CoV-2 vaccination may increase the risk of developing an autoimmune disease such as lupus erythematosus, rheumatoid arthritis, thrombocytopenia^2^ and uveitis inflammatory disorder.^3^ To the best of our knowledge, researches investigating the onset of Acquired Hemophilia A (AHA) and sarcoidosis following SARS-CoV-2 vaccination were scarce.

Acquired hemophilia A is a rare autoimmune disease caused by the appearance of neutralizing anti-factor VIII (FVIII) autoantibodies in individuals with no previous history of bleeding.^4^ Most often, patients can simultaneously be affected by multiple diseases, including, malignancies, and several autoimmune disorders such as systemic lupus erythematosus, autoimmune thyroiditis, rheumatoid arthritis, or pemphigus.^5^ Among 50% of cases, the underlying triggering cause remains unknown. For this reason, an extensive history of immunological conditions, drugs, and the wide range of linked medical issues that predispose the patient to acquired hemophilia should be gathered.^6^

The pathophysiology of AHA is not well elucidated. However, T-cells are thought to have a role, as are several genetic polymorphisms.^7^ The development of autoantibodies and vaccination has long been associated. Due to antigenic mimicry and the stimulation of dormant autoreactive T and B-cells, it has been suggested that vaccination may trigger an autoimmune reaction.^8^ In this context, Schultz NH and collaborators have demonstrated that antibodies against COVID-19 spike glycoproteins can react with host peptide protein sequences that are structurally similar to the membranes of coagulation factors or red blood cells.^9^ This can lead to an autoimmune mechanism in the cases of acquired hemophilia A.^10^ In these circumstances, numerous cases have reported that SARS-CoV-2 vaccination can be potential triggers for AHA in healthy patients.^11^, ^12^, ^13^, ^14^

Sarcoidosis is another inflammatory disorder that can be observed following SARS-CoV-2 vaccination. It is a multisystem disorder of unknown etiology. Some cases are linked to autoimmune involvement, self-antigens, and genetic factors that cause inflammation. It has been reported that SARS-CoV-2 vaccination can induce inflammatory phenomena like sarcoidosis.^2^ Few new cases of sarcoidosis have been reported after COVID-19 infection, and even after SARS-CoV-2 vaccination.^15^ COVID-19 vaccine-associated sarcoidosis can be explained not only by abnormal regulation of the immune system^16^ but also by an impairment of the angiotensin pathway.^17^ In this context, it has been reported that spike protein binds to the angiotensin II receptor which allows the virus to enter the cell.^18^ Evenly, immune dysregulation and inflammatory activation can be caused by COVID-19 vaccine adjuvants.^19^ Reviewing the literature, the authors found two healthy cases which have developed systemic sarcoidosis following SARS-CoV-2 vaccination. These two subjects have received the booster doses of the ribonucleic acid vaccine and symptoms started after the first dose. The main symptoms of these cases were fever, fatigue, dyspnea, and dry cough.^20^^,^^21^ Previously, it has been reported a case of sarcoidosis exacerbation presenting after SARS-CoV-2 vaccination.^22^

In conclusion, several case reports have suggested vaccines may trigger an autoimmune reaction. However, it should nonetheless be highlighted the overwhelming benefits of SARS-CoV-2 vaccination and underling that advantages can exceed potential adverse reactions in order to prevent COVID-19 morbidity and mortality. Therefore, mechanisms showing the association between vaccines, autoimmunity phenomena, and inflammatory manifestations remain poorly understood and need to be further investigated. For this reason, clinicians need to be attuned to the uncommon chance of the possible flare-ups of underlying diseases such as AHA and sarcoidosis.

## Data availability statement

All data generated and analyzed in this present study are available from the corresponding author upon reasonable request.

## Funding

No specific grant was given to this research.

## Declaration of Competing Interest

The authors declare no conflicts of interest.
